# Supplementary material for: The Functional Characterization of Carboxylesterases Involved in the Degradation of Volatile Esters Produced in Strawberry Fruits
Source: Int J Mol Sci. 2022 Dec 26;24(1):383. doi: 10.3390/ijms24010383 (PMC9820763; doi:10.3390/ijms24010383)
Supplement: Supplementary file 1 [file ijms-24-00383-s001.zip › Supplementary Figures.pdf]

Supplymentary Figure

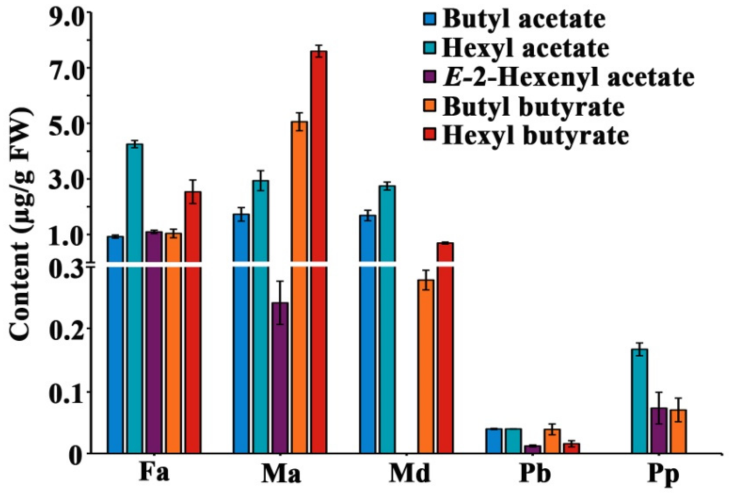

Figure. S1 The content of main volatile esters in five fruits

Note: Fa, strawberry; Ma, banana; Md, apple; Pb, pear; Pp, peach

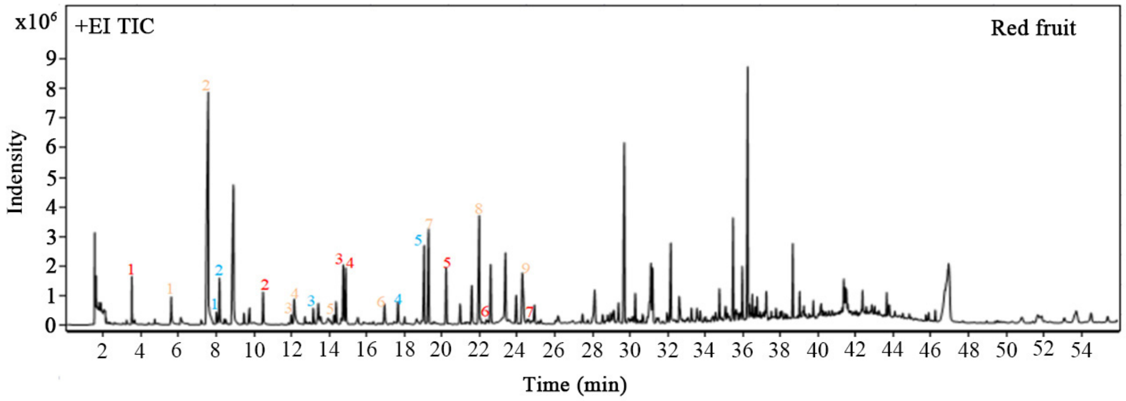

Figure. S2 Ion chromatogram of strawberry extract

Note：Red numbers represent esters, blue numbers represent alcohols and yellow numbers represent aldehydes.
